# A centralised public information resource for randomised trials: a scoping study to explore desirability and feasibility

**DOI:** 10.1186/1472-6963-5-39

**Published:** 2005-05-24

**Authors:** Anne L Langston, Marion K Campbell, Vikki A Entwistle, Zoë Skea

**Affiliations:** 1 Health Services Research Unit, University of Aberdeen, Aberdeen, UK

## Abstract

**Background:**

There are currently several concerns about the ways in which people are recruited to participate in randomised controlled trials, the low acceptance rates among people invited to participate, and the experiences of trial participants. An information resource about on-going clinical trials designed for potential and current participants could help overcome some of these problems.

**Methods:**

We carried out a scoping exercise to explore the desirability and feasibility of establishing such a resource. We sought the views of a range of people including people who were considering taking part in a trial, current trial participants, people who had been asked but refused to participate in a trial, consumer group representatives and researchers who design and conduct trials.

**Results:**

There was broad-based support for the concept of a centralised information resource for members of the public about on-going and recently completed clinical trials. Such an information resource could be based on a database containing standardised information for each trial relating to the purpose of the trial; the interventions being compared; the implications of participation for participants; and features indicative of scientific quality and ethical probity. The usefulness of the database could be enhanced if its search facility could allow people to enter criteria such as a disease and geographic area and be presented with all the trials relevant to them, and if optional display formats could allow them to view information in varying levels of detail. Access via the Internet was considered desirable, with complementary supported access via health information services. The development of such a resource is technically feasible, but the collation of the required information would take a significant investment of resources.

**Conclusion:**

A centralised participant oriented information resource about clinical trials could serve several purposes. A more detailed investigation of its feasibility and exploration of its potential impacts are required.

## Background

The rigorous evaluation of health care interventions requires potential users of these interventions to participate in clinical trials to assess their effectiveness. There are currently various concerns about both the small proportion of eligible people who participate in randomised controlled trials (RCTs) and the experiences of participants [1,2]. Inadequate information provision is a contributing factor to several of these concerns. For example, some people who would like to enter RCTs are not invited to do so, and struggle to identify trials for which they would be eligible [3]. Problems have been identified with the quality of information given [4]. Thus some people who are invited to participate in RCTs do not develop an adequate understanding to make a well-informed decision to participate or not [5,6]. People who agree to participate in trials sometimes struggle to make sense of their participation [7], may not know what to do if they identify questions during the course of the trial, and are often disappointed at the lack of information they are given about the progress or results of the trials [8].

An information resource about ongoing clinical trials designed for potential and current participants could help overcome some of these problems. It could serve as a source of additional, semi-independent information for people who have been invited to participate in trials, and it could help people to identify trials in which they might participate. If kept up to date, it could serve as a source of information about the progress and results of particular trials.

A number of trial registers have been established in recent years (for example, *meta*Register of Controlled Trials , UKCCCR National Register of Cancer Trials ), and there has been increasing pressure for requirements that all clinical trials be registered in the public domain. [9]. Trial registers are undoubtedly helpful in terms of getting information about planned, ongoing and completed trials into the public domain and reducing the problems associated with underreporting of trials. However, the current registers are not comprehensive [10-12]. They were not designed to be easily accessible or useful to general public audiences and none were designed specifically to meet the information needs of members of the public as potential or current trial participants. It can be difficult to identify which registers are appropriate for particular purposes [12,13], and it is hard to search across registers because they do not organise and present information in a standardised way [3,13].

In this paper we report on a scoping exercise undertaken for Consumers in NHS Research (now known as INVOLVE) to assess the desirable features and feasibility of developing and providing an information resource about ongoing clinical trials for potential and current participants [14].

## Methods

We recognised that people who had played different roles in relation to trials would have different perspectives on information needs relating to clinical trials. Reflecting this, we sought information and opinions from a range of people from across the UK that included: people seeking to participate in or considering participating in a trial; current and recent trial participants; representatives of national patient interest/support groups; and researchers involved in the design and conduct of trials. People who were seeking to participate and considering participating in a trial were identified through condition-specific consumer networks, in particular the National Association for the Relief of Paget's Disease (a trial investigating the treatment of Paget's disease was about to start at the time we conducted this scoping exercise). Current and recent trial participants were identified at three meetings of health care consumers and advocates. Representatives of consumer groups were selected from those patient interest/support groups with a known interest in trials (identified primarily by Consumers in NHS Research). Researchers involved in the design and conduct of trials were selected from the UK MRC trial managers network and individuals known to the research team. Several of the consumer advocates and researchers had current or recent personal experience of participating (or declining to participate) in clinical trials. We accepted their contributions from the perspective of trial participants, because the time frame of our scoping exercise did not permit us to obtain ethics committee approval to identify and approach current participants via trial offices.

We used semi-structured interviews, focus groups and e-mail discussion lists to explore the kinds of information that people want and need when considering whether or not to participate in a particular trial, and the ways they might access and use this information. We asked people to discuss the feasibility and appropriateness of 'star rated' information on the quality of particular aspects of ongoing trials, and the provision of generic guidance about how to interpret and appraise information about trials. We also asked researchers and consumer representatives what they thought made for a good quality trial and what issues might arise from an attempt to provide standardised information about ongoing randomised clinical trials in a publicly accessible participant-orientated information resource.

We used topic guides to support semi-structured interviews and focus group discussions. An example of the topic guide used with potential trial participants is included [see Additional file 1]. Most interviews were audio-taped and transcribed in full. If audio-recording was not possible, members of the research team took detailed notes. Analysis of transcripts, discussion notes and e-mails was carried out using a modified 'charting' approach [15]: to facilitate the identification of the range of views about particular issues, the points made by each participant were summarised under key headings that reflected the main areas of questioning.

Over the course of the scoping exercise we formally interviewed 25 individuals on a one-to-one basis including:

• nine potential trial participants;

• six representatives from patient interest /support groups;

• nine researchers (including trial principal investigators, trial managers and a research nurse); and

• one pharmaceutical/medical devices industry representative.

In addition, the views of 24 consumer advocates were elicited in focus groups held at national meetings of consumers and advocates, and nine consumer representatives contributed comments via e-mail. Several of the consumer advocates and researchers had personal experience of participating (or declining to participate) in clinical trials.

## Results

### Information elements required by potential trial participants

**Figure 1:**
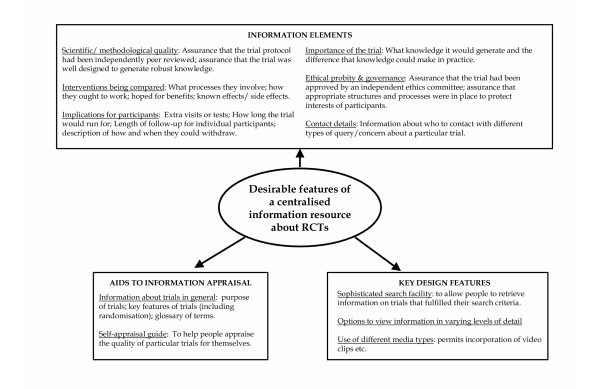
The features identified as desirable for a centralised information resource about randomised controlled trials.

Our informants identified a range of information needs of potential trial participants. We grouped these into the following categories: (a) the interventions being compared; (b) the implications of trial participation for participants; (c) the scientific or methodological quality of a trial; (d) the ethical probity and governance of a trial; and (e) contact details (Figure 1).

#### Interventions being compared

There was a consensus that potential trial participants need to know about the 'new' intervention being tested, its potential risks, and how it is known to compare with the current "standard" intervention and any other interventions with which it is compared in the trial. Information about the availability of relevant interventions outwith the trial was also mentioned as important.

#### Implications for participants

Information needs relating to implications for participants included practical issues such as the timescales for the trial interventions and follow-up, and the rationale for, and number of extra visits or investigations associated with the trial. Several informants highlighted the need for information about whether and how people could withdraw from or join the trial at a later date. They also thought that participants should be told whether or how they could gain access to data about their own outcomes and the trial findings, and about whether and how participants would be told if a trial was stopped early for any reason.

#### Scientific or methodological quality, and ethical probity and governance

There was a consensus that people should be able to be confident about the quality of any trial they might agree to participate in, but it was widely recognised that relatively few people would currently be able to judge the scientific quality or ethical probity of a trial for themselves on the basis of the information they are typically given for recruitment purposes. The provision of information about key features of trial quality (and/or about quality assessment) was thought desirable for a centralised resource.

#### Contact details

Several respondents from all categories mentioned that potential trial participants might benefit from the option to talk to someone before deciding whether or not to participate in a trial, and possibly also during the trial. The case was regularly made that two-way interaction was key to understanding information, as any misconceptions could be corrected and personal concerns could be addressed. For people who had been invited to participate in a particular trial, the options suggested were:

• having a chance to speak with a member of the trial team (preferably someone who is not related directly to a person's normal care) – someone who can explain things like randomisation;

• having a chance to talk to an independent person (i.e. someone not associated with the trial) e.g. GP, consumer advocate, voluntary health organisations, advice services; and

• having contact details, so know whom to contact if concerned about anything while participating in the trial.

People who were themselves looking for trials in which they might participate would be likely to need:

• contact details for initial enquiries about the specific trial and (possibly subsequently) local recruiting centres/clinicians; and possibly

• an information helpline staffed by people who could offer general information, answer general questions about trials, and help explain information provided about specific trials.

However, the capacity of trialists or trial centres to handle unsolicited enquiries was limited, they might be reluctant to have their contact details 'advertised' in the context of an information resource that promised help to people identify trials in which they might participate.

### Aids to information appraisal

As indicated in the methods section, we explored two approaches that might help people evaluate information about trials and assess their quality: (a) a quality or "star" rating scheme in which key features of trials were allocated one- to three- star ratings; and (b) a guide to help people appraise trials for themselves.

Informants registered some strongly felt concerns about the quality ratings option. These included doubts about the feasibility of producing valid and reliable ratings across a diverse range of trials, and fears that particular ratings were likely to be contested by trial sponsors and trialists, which could create a lot of 'hassle' and work for the resource producers.

Despite the limitations of a quality ratings scheme, there was general agreement that it would be useful to indicate that key quality assurance features were in place for particular trials. The most obvious features suggested were that a trial had been through an independent peer review process (a check on scientific quality) and that it had been assessed and approved by an independent ethics committee (a check on ethical probity).

There was widespread support for the provision of a general information section within any centralised resource that could: explain the purpose of clinical trials; highlight key features and indicate points to look out for in clinical trials; provide a glossary of commonly used terms; and identify links to relevant organisations. The inclusion of a 'guide' to help people appraise the quality of a trial for themselves was also thought appropriate. This guide could be based on existing checklists developed for peer reviewers, ethics committees and consumer representatives who appraise trials, but modified for more general public use.

### Key design features of a centralised resource

All informants supported the basic concept of a centralised information resource about clinical trials. This could be built using a database structure with a standard set of information elements for each trial reflecting the key areas outlined above. Respondents identified a variety of design features that could potentially enhance the usefulness of the information within the resource, including:

• A sophisticated search facility that would allow people to enter their personal details (e.g. health condition, age, gender, geographical location etc), and retrieve information on trials that fulfilled their search criteria.

• Options to view particular types of information in varying levels of detail.

• The use of a variety of media within or as a complement to the resource (for example, diagrams or video-clips to illustrate and explain trial procedures or the interventions being compared in particular trials

### Potential models for providing a centralised information resource

There was widespread support for the provision of this centralised information resource on the Internet, but a clear recognition that some people would not be able to access the information effectively via this route. Opportunities for supported access via outlets in health care settings, public libraries or other community centres would also be useful. Health information services might usefully serve as valuable 'intermediaries', adding value to the resource by helping people to appraise the information and interpret it for their own use.

## Discussion

One of the strengths of our study is that we canvassed views from a range of perspectives. We identified a broad-base of support for the concept of a centralised, publicly accessible, participant-oriented information resource about ongoing clinical trials. We also identified several concrete suggestions for the content and design of such a resource. However, time constraints prevented us from exploring the views of a larger sample of people, and from considering the issues we identified in more depth. We acknowledge in particular that further research is needed to explore the opinions of a wider spectrum of the 'general public' as potential users of a centralised resource for information about clinical trials, and to consider the potential implications of presenting particular elements of information and in particular ways. We also acknowledge that the potential trial participants interviewed for this study were mostly people with a chronic condition. Their perspectives may differ from potential trial participants with acute health problems. However, individuals with acute conditions were canvassed for their views as current or recent trial participants. We do not claim to have identified all possible information requirements, but are confident that we have identified the main ones.

In theory, a centralised information resource about clinical trials would have two main audiences in terms of trial participants: those who have been invited to participate in a trial and want to find out more about it, and those who have not been invited to participate in a trial, but would like to identify any ongoing trials for which they might be eligible. If the resource included updated reports on trial progress, it may also be useful to a third group of people who are currently participating in trials who want more information about the trials in which they are participating

It is unclear whether and to what extent a centralised information resource would achieve the desired aims of improving recruitment rates, and people's experiences of information provision relating to trial recruitment and participation, nor whether it would have any unwanted effects. It is not clear to what extent a publicly accessible resource might increase demand for participation in trials, nor what the implications of such demands might be. At the moment, some health care providers are not in a position to offer trial participation to their service users. Increased public awareness of particular trials might place a variety of extra burdens on health care systems, for example if patients start demanding access to particular trials and/or changing health care providers in order to gain access to them.

Other potential disadvantages of making information about trials more easily accessible should be explored. For example, breaches of privacy might become a problem if the knowledge that someone is eligible to participate in a particular trial (which is often readily obtained by third parties who observe markers on medical records or witness the receipt of mailings from a trial team) could quickly lead to the revelation of information about personal health status or health care experiences because trial eligibility criteria would be easily ascertained. Public access to trial progress reports might, depending on the nature of information provided, influence people's self-reporting of outcome status and willingness to continue to participate in a trial. Issues such as these require further consideration and investigation.

While the development of the database framework, search engine and the features identified above as desirable are technologically feasible, there are several potential barriers to the development of a participant-oriented central information resource about clinical trials. Current trial registers do not contain all the information elements that potential participants might find useful. The process of gathering, checking and updating the requisite information would be logistically challenging and time consuming, and would require considerable and sustained investment. Any attempt to include trial progress reports to the database would significantly add to the logistical challenges. However, recent trends towards increasing disclosure of information about trials, including commitments to openness from some pharmaceutical companies, will facilitate the acquisition of relevant information.

## Conclusion

A centralised participant-oriented information resource about randomised trials could offer many benefits. The development of such a resource would require considerable investment, but the support for the basic concept that is evident among trial participants, consumer advocates and trialists suggest that it warrants further investigation and evaluation.

## Abbreviations

UKCCR United Kingdom Coordinating Committee on Cancer Research

RCT Randomised Controlled Trial

## Competing interests

The author(s) declare that they have no competing interests.

## Authors' contributions

All authors contributed equally to the design and execution of the study. MKC was principal investigator. ALL wrote the first draft of the manuscript. All authors contributed to the development of subsequent drafts, and read and approved the final manuscript.

## Pre-publication history

The pre-publication history for this paper can be accessed here:



## Supplementary Material

Additional File 1 2001. Example Topic Guide. Click here for file
