# Supplementary material for: A centralised public information resource for randomised trials: a scoping study to explore desirability and feasibility
Source: BMC Health Serv Res. 2005 May 24;5:39. doi: 10.1186/1472-6963-5-39 (PMC1168894; doi:10.1186/1472-6963-5-39)
Supplement: Additional File 1 — 2001. Example Topic Guide. [file 1472-6963-5-39-S1.doc]

**
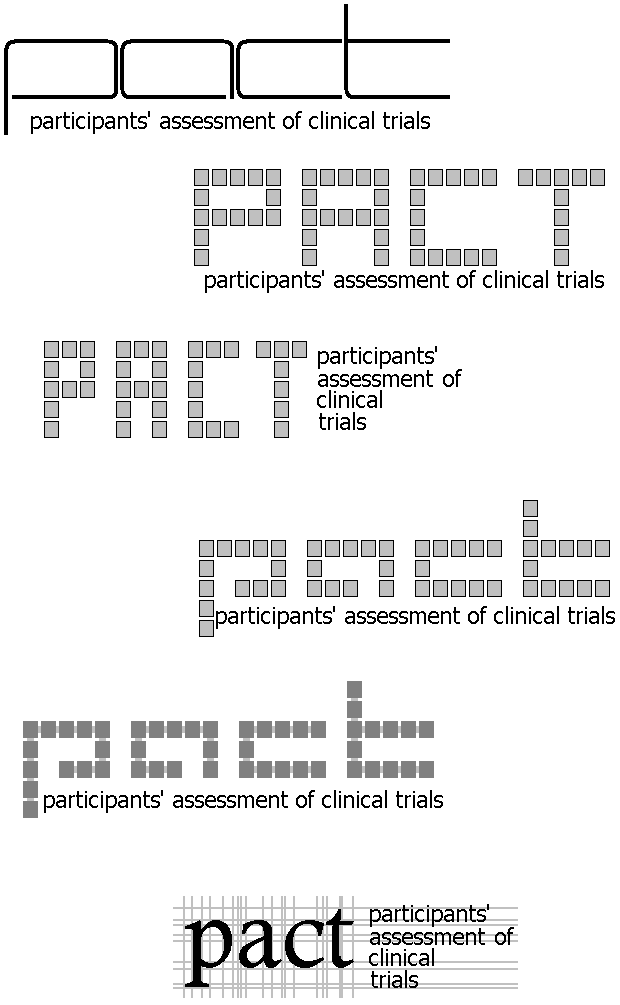
**

### PACT TOPIC GUIDE

**FOR INDIVIDUALS INTERESTED IN TAKING PART IN A CLINICAL TRIAL**

## Interview

- Investigate background of each person (e.g. A health problem/ condition you have or have had? What is it/ how does it affect you? Any treatments you use or have used?)
- “Have you been asked to participate in a trial before, have you ever thought about/participated in a trial before? If YES, can you tell me about that?” (If they have received information leaflet before, ask for views on the information received.)
- Ask individual to imagine there are several trials ongoing for their condition. Each trial compares (e.g. two treatments).
- Explain what is meant by a trial (e.g. If they took part in a trial this would mean that they would be allocated to either one treatment or the other, by a computer, and that no one could choose which treatment they received. This is done to ensure that similar types of people receive both treatments.)
- “Your doctor wondered if you might like to get involved in one of the trials. What would you want to know as you consider whether or not you would take part? What would most influence your decision to take part?”

Probe and check

- “If you were approached about a trial you would be given an information leaflet. What features would you like to see in any information leaflet? What do they think makes information good or bad, and what would make them decide to join the trial?

Probe and check

- Is there anything they **wouldn’t** want to know?

Probe and check

- Where would they look for the types of information spoken about?
- Where would they **like** to be able to find it?
- Explain that we are trying to decide where best to provide this sort of information. Suggest options e.g. via electronic register, reference book in library, central telephone information line e.g. NHS 24. Ask which they would find best.
- Discuss vignettes

(Option 1 – list of facts; option 2 – rating of facts; option 3 – facts and rating combined)

Explain that none of the options provided is comprehensive, but gives a flavour of how information could be presented in different ways. Ask which one they preferred and explore reasons why

Probe and check

- If a guide were available to help them evaluate a trial themselves, would they use it? Would they find it useful?
- Are any other areas that we should be covering in our study?
